# Pediatric training and practice of Canadian chiropractic and naturopathic doctors: a 2004–2014 comparative study

**DOI:** 10.1186/s12906-017-2024-5

**Published:** 2017-12-01

**Authors:** Antony Porcino, Leslie Solomonian, Stephen Zylich, Brian Gluvic, Chantal Doucet, Sunita Vohra

**Affiliations:** 1 Department of Pediatrics, CARE Program, Faculty of Medicine, University of Alberta, Edmonton, Canada; 2 Canadian College of Naturopathic Medicine, Toronto, Canada; 3 Canadian Memorial Chiropractic College, Toronto, Canada; 4 Boucher Institute of Naturopathic Medicine, New Westminster, Canada; 5 Département de Chiropratique, Université du Québec à Trois-Rivières, Trois-Rivières, Canada; 6 Department of Public Health Sciences, School of Public Health, University of Alberta, Edmonton, Canada; 7 Department of Pediatrics, CARE Program, Faculty of Medicine and Dentistry, Suite #1702, College Plaza, 8215 112 St NW, Edmonton, AB T6G 2C8 Canada

**Keywords:** Pediatrics, Integrative medicine, Complementary medicine, Survey, Naturopathic, Chiropractic

## Abstract

**Background:**

To assess chiropractic (DC) and naturopathic doctors’ (ND) knowledge, attitudes, and behaviour with respect to the pediatric patients in their practice.

**Methods:**

Cross-sectional surveys were developed in collaboration with DC and ND educators. Surveys were sent to randomly selected DCs and NDs in Ontario, Canada in 2004, and a national online survey was conducted in 2014. Data were analyzed using descriptive statistics, t-tests, non-parametric tests, and linear regression.

**Results:**

Response rates for DCs were *n* = 172 (34%) in 2004, *n* = 553 (15.5%) in 2014, and for NDs, *n* = 171 (36%) in 2004, *n* = 162 (7%) in 2014. In 2014, 366 (78.4%) of DCs and 83 (61%) of NDs saw one or more pediatric patients per week. Pediatric training was rated as inadequate by most respondents in both 2004 and 2014, with most respondents (*n* = 643, 89.9%) seeking post-graduate training by 2014. Respondents’ comfort in treating children and youth is based on experience and post-graduate training. Both DCs and NDs that see children and youth in their practices address a broad array of pediatric health concerns, from well child care and preventative health, to mild and serious illness.

**Conclusions:**

Although the response rate in 2014 is low, the concerns identified a decade earlier remain. The majority of responding DCs and NDs see infants, children, and youth for a variety of health conditions and issues, but self-assess their undergraduate pediatric training as inadequate. We encourage augmented pediatric educational content be included as core curriculum for DCs and NDs and suggest collaboration with institutions/organizations with expertise in pediatric education to facilitate curriculum development, especially in areas that affect patient safety.

## Background

Complementary therapies encompass a broad array of modalities, such as natural health products (NHPs, such as nutritional supplements and herbal medicines), spinal manipulation, massage, and acupuncture [1]. Rates of use vary from 9% to 83% depending on the population studied and definitions used, with higher rates in patients with chronic, recurrent, or serious illness conditions [2, 3].

At least 160 studies describing pediatric complementary therapies use have been documented, [4] however the vast majority have been conducted in hospitals or conventional medical outpatient clinics. Because most patients do not report complementary therapies use to their physician, [5] studies conducted in conventional medical settings may not sufficiently describe pediatric complementary therapy use.

To investigate the demographics, knowledge, attitudes, and behaviours of complementary therapy practitioners regarding pediatric patients in their practice, and to evaluate any changes over time, we conducted two surveys. In 2004, we surveyed chiropractic and naturopathic doctors (DCs and NDs, respectively) in Canada’s largest province. In 2014, we conducted a national survey of DCs and NDs.

## Methods

### Survey development

In accordance with accepted survey methodology, [6] two surveys (DC, ND) were developed based on a literature review and discussion with DC and ND experts. Some questions were adapted with permission from the published literature to yield comparable data [7, 8]. Key survey domains included: (1) practitioner demographics (e.g., gender, year of graduation, clinical practice details); (2) practitioner pediatric training (e.g., under- and post-graduate education); and (3) practitioner treatment of and attitudes to selected common pediatric issues. Only practitioners seeing one or more pediatric patients per week were invited to complete the section on practitioner attitudes and treatments. To assess practitioner behaviour, all survey respondents assessed three case scenarios involving ill children. Most survey questions were identical for both professions, with some questions modified according to the profession’s practices. The 2014 survey used the 2004 questions, with some modifications to assess additional detail. In both years, surveys were piloted prior to launch.

### Participant recruitment

In 2004, a postage-paid-return paper survey was used with accepted methodology [9]. DCs and NDs practicing in Ontario, Canada were identified from educational institution lists. Surveys were sent to all 453 NDs and a random sample of 500 of the 3500 DCs. To maximize response rate, surveys included introductory letters on letterhead from the appropriate educational college.

The 2014 survey used an online survey system using accepted methodology [10]. To maximize response rate, Canadian DCs and NDs were sent emails with a link to the questionnaire via their provincial or national association, regulatory body, or educational college.

For all surveys, if respondents indicated they saw at least one pediatric patient per week, all data were included for analysis. If the respondent reported not seeing even one pediatric patient per week, only demographic, training, and case study responses were assessed.

### Analysis

The primary analysis was descriptive (means and standard deviations for continuous data, frequencies for categorical data). Secondary analyses involved logistic regression and t-tests for continuous variables, and chi-square for categorical data. All comparisons were 2 tailed and considered statistically significant at *p* < .05. Statistical tests were done both within and between professions each year (2004 and 2014). Because chiropractic and naturopathic educators stated that schools had updated the pediatric curriculum in the last 5 years, post-hoc analysis of results regarding training adequacy, satisfaction, and number of pediatric clients using a five-year split point (2009) were also done.

The surveys received ethical approval from the University of Alberta Human Research Ethics Board.

## Results

Response rates by profession were 34% (172/500) for DCs in 2004 and 15.5% (553/3564) in 2014, 36% (161/453) for NDs in 2004 and 7.1% (162/2280) in 2014.

### Demographics

In both years, DCs had a significantly lower proportion of female respondents (34.8% in 2004, 54.0% in 2014, *p* < .001) relative to the NDs (79% in 2004, 85% in 2014, *p* < .001) (DC relative to ND: *p* < .001). The average age of responding DCs increased from 40.9 years (SD 9.1) to 43.8 years old (SD 11.1) (*p* < .001), while the average age of responding NDs did not change (38.7 (SD 8.9) to 37.6 (SD 8.6)). Practitioner location in different-sized communities was not significantly different within or between professions in 2004. Slightly more responding NDs lived in larger communities in 2014 (*p* = .025). There were no significant differences within or between provider groups with regards to graduate-level degrees in 2004; DCs held significantly more graduate degrees than NDs in 2014 (DC: 7.2%; ND: 3%; *p* = .001).

In 2004, 10.4% of DC and 19% of ND respondents did not see one or more pediatric patients per week. In 2014, those percentages were 21.6% of DC and 40% of ND respondents. For both years, there were no statistically significant differences in age, gender, or years of or place of practice between those who reported seeing pediatric patients and those who did not. In 2014, DC practitioners seeing at least one pediatric patient per week (Table 3) reported significantly more patients (114.9 versus 51.9, *p* < .001); both DCs and NDs seeing pediatric patients had significantly more practice hours per week (DCs: 29.2 versus 24.6, *p* = .002; NDs: 21.9 versus 15.6, *p* = .046). Demographic details are in Table 1.

**Table 1:** Practitioner characteristics

		Chiropractic Doctors (DC)			Naturopathic Doctors (ND)			DC vs. ND
		2004	2014	2004 vs. 2014	2004	2014	2004 vs. 2014	
Gender (female). n (%)		57 (35)	216 (54.0)	***p < .001*** ^**e**^	127 (79)	89 (85)	***p*** **< .001** ^**e**^	**2004:** ***p*** ** < .001** ^**b**^ **2014:** ***p*** **< .001** ^**b**^
Post-secondary Education. n (%)	BA or BS	109 (81)	359 (76.5)	*p* = .56^b^	120 (87)	139 (90)	*p* = .42^b^	2004: *p* = .23^b^ 2014: *p* < .001^b^
	MSc or PhD	9 (7)	34 (7.2)		9 (7)	5 (3)		
	Other	17 (13)	76 (16.2)		9 (7)	10 (7)		
Age. mean (SD)		41 (9)	44 (11.1)	***p < .001*** ^**c**^	39 (10)	38 (9)	*p* = .62^**c**^	2004: *p* = .16^**c**^ **2014:** ***p*** **< .001** ^**c**^
Years in practice. mean (SD)		13 (10)	17 (10.9)	***p < .001*** ^**c**^	8 (7)	9 (7)	*p* > .99^**c**^	**2004:** ***p*** ** < .001** ^**c**^ **2014:** ***p*** **< .001** ^**c**^
Size of community of practice. n (%)	Rural (<10,000)	28 (17)	50 (12.3)	*p* = .085^b^	13 (7)	6 (6)	*p* = .83^b^	2004: *p* = .15^b^ **2014:** ***p*** ** = .025** ^**b**^
	Small town (10,000–49,999)	28 (17)	76 (18.8)		27 (17)	15 (14)		
	Medium-sized city (50,000–99,999)	32 (19)	50 (12.3)		33 (20)	22 (20)		
	Suburban outside a metro area	13 (8)	51 (12.6)		18 (11)	9 (8)		
	Metropolitan area (>100,000)	70 (41)	178 (44)		78 (48)	56 (52)		
Hours of practice per week. mean (SD) / range		29 (9) 1–50	28 (11) 1–65	*p* = .98^c^	24 (10) 1–50	18 (13) 1–44	***p < .001*** ^**c**^	**2004:** ***p*** ** < .001** ^**c**^ **2014:** ***p*** **< .001** ^**c**^
Patients seen per week. mean (SD) / range		131 (81) 1–550	101 (82) 1–600	***p < .001*** ^**c**^	23 (18) 1–120	18 (24) 1–60	***p*** **= .05** ^**c**^	**—** ^**d**^
Sees pediatric patients. n (%)		146 (89.2)	366 (78.4)	***p < .001*** ^**a**^	125 (89)	83 (60)	***p < .001*** ^**a**^	2004: *p* = .94^a^ **2014:** ***p*** **< .001** ^**a**^
2014 only	Sees pediatric patients	yes (*n* = 366)	no (*n* = 101)		yes (*n* = 83)	no (*n* = 53)		
	Hours of practice per week. Mean (SD)	29.2 (12.1)	24.6 (13.2)	***p = .002*** ^**c**^	21.9 (11.8)	15.6 (20.4)	***p = .046*** ^**c**^	***p < .001*** ^**e**^
	Patients seen per week. Mean (SD)	114.9 (82.1)	51.9 (48.1)	***p < .001*** ^**c**^	14.6 (25.5)	23.1 (26.5)	*p* = .06 ^**c**^	**—** ^**d**^

a: z-score. b: chi-squared tests. c: t-tests. d: not examined given difference in DC–ND clinical visit time. e: ANOVA. Bold items are statistically significant

### Pediatric training

#### Lectures

The majority of DCs (55.0% in 2004, 55.8% in 2014) and NDs (63% in 2004, 64% in 2014) reported receiving one semester or more of lectures about pediatrics during their training (all training data are in Table 2). In 2004, only 15.8% of DCs and 27% of NDs rated their training as somewhat or very adequate. To better understand perceived areas of deficiency, in 2014, adequacy of pediatric training was split into training about diagnosis and treatment. In 2014, 31.1% of DCs reported their diagnostic training and 24.2% reported their treatment training as somewhat or very adequate. In 2014, 33% of NDs reported their diagnostic training and 37% reported their treatment training as somewhat or very adequate. In both years, DCs consider their pediatric training less adequate compared to NDs (*p* < .001).

**Table 2:** Pediatric training

		Chiropractic Doctors (DC)			Naturopathic Doctors (ND)			DC vs. ND^a^
		2004	2014	2004 vs. 2014^a^	2004	2014	2004 vs. 2014^a^	
Amount of lecture-based training. n (%)	None	5 (3)	7 (0.9)	***p*** **< .001**	4 (3)	4 (2)	***p*** **= .002**	2004: *p* = .23 **2014:** ***p*** **< .001**
	Occasional	36 (21)	321 (43.3)		20 (13)	76 (32)		
	1 semester	75 (44)	29 (3.9)		79 (50)	106 (45.		
	1 year	19 (11)	385 (51.9)		20 (13)	45 (19)		
	Don’t recall	21 (12)	0 (0)		26 (17)	4 (2)		
Rating of lecture-based training.^b^ n (%)	Very adequate	5 (3)	38 (7.1)	*p* = .33	5 (3)	13 (8)	*p* = .98	2004: *p* = .35 2014: *p* = .45
	2	21 (13)	111 (20.6)		36 (23)	43 (27)		
	3	67 (41)	174 (32.3)		51 (33)	57 (36)		
	4	41 (25)	127 (23.6)		39 (25)	30 (19)		
	Not adequate	30 (18)	89 (16.5)		22 (14)	15 (9)		
Amount of hands-on training. n (%)	None	89 (52)	238 (49.2)	*p* = .31	33 (21)	36 (23)	*p* = .16	**2004:** ***p*** **< .001** **2014:** ***p*** **< .001**
	Minimal	64 (37)	161 (33.3)		106 (67)	93 (58)		
	Significant	7 (4)	41 (8.5)		8 (5)	20 (13)		
Rating of hands-on training. n (%)	Very adequate	5 (3)	23 (4.8)	*p* = .15	1 (1)	12 (8)	*p* = .086	**2004:** ***p*** **= .042** 2014: *p* = .174
	2	12 (7)	52 (10.8)		15 (10)	16 (11)		
	3	26 (16)	88 (18.3)		42 (27)	36 (24)		
	4	34 (21)	124 (25.8)		45 (29)	48 (32)		
	Not adequate	82 (52)	193 (40.2)		51 (33)	36 (24)		
Comfort level treating children.^c^ n (%)	Very comfortable	61 (36)	311 (62.4)	***p*** **< .001**	45 (28)	72.6 (48)	***p*** **< .001**	2004: *p* = .55 **2014:** ***p*** **= .006**
	2	48 (28)	82.4 (16.5)		53 (33)	44.4 (30)		
	3	33 (19)	47.2 (9.5)		34 (21)	15.6 (10)		
	4	20 (12)	26.8 (5.4)		26 (16)	9.2 (6)		
	Not comfortable	9 (5)	30.2 (6.1)		4 (3)	7.6 (5)		

a: chi-squared tests. b: each 2014 adequacy value is an average of the diagnostic and treatment adequacy scores, to enable comparison with 2004. c: comfort by age category was only assessed in 2014; each 2014 comfort value is the average combined comfort scores for the 5 age categories, to enable comparison with 2004. Bold items are statistically significant

#### Hands on

About half of DCs (51.7% in 2004, 49.2% in 2014) and one fifth of NDs (21% in 2004 and 23% in 2014) reported they received no hands-on clinical pediatric training. Only a minority of practitioners felt their hands-on pediatric training was adequate (somewhat or very) for their needs: DCs: 10.6% in 2004, 15.6% in 2014; NDs: 10% in 2004 and 19% in 2014.

#### Additional training and training impacts

While 78% of respondents trained before 2009 see one or more pediatric patients per week, only 55.4% of respondents trained between 2009 and 2014 report seeing pediatric patients. Over half of both DCs and NDs pursued additional pediatric training after graduation from their programs: DCs: 61.0% in 2004, 93.1% in 2014; NDs: 51% in 2004, 79% in 2014. By 2014, 89.9% (*n* = 643) of all respondents had sought additional training.

Despite respondent data about their undergraduate pediatric training and its perceived inadequacy, the majority of providers were comfortable caring for pediatric patients, increasing from an average 62% (somewhat or very comfortable) in 2004 to 78% in 2014 (in 2014, degree of comfort was assessed by age category; an average is used for comparison with 2004). In 2014, respondents indicated that the older the pediatric patient, the more comfortable (somewhat or very) the practitioners are with patient care, increasing from an average of 62% for newborns, to 98% for adolescents. Logistic regression indicated that for DCs in 2014 the primary predictor for comfort in treating pediatric patients was years of experience (very comfortable: odds ratio (OR) 1.35; 95% confidence interval (CI): 1.14–1.59). For NDs in 2014, the only predictor for comfort in treating pediatric patients was having post-graduate training in pediatric care (very comfortable: OR 43.5; 95% CI: 3.1–200; somewhat comfortable: OR 15.4; 95% CI: 1.7–142.9).

Although the pediatric educators on our research team described enhancements to pediatric education 5 years ago, analysis did not reveal any improvement in newer practitioners’ comfort in pediatric care. In fact, the reverse was found: provider comfort (somewhat or very comfortable) was significantly lower for those trained post-2009 (*p* = .019).

Further data analysis was restricted to practitioners who reported seeing at least one pediatric patient per week (DCs: 2004, *N* = 169; 2014 *N* = 339. NDs: 2004, *N* = 156; 2014 *N* = 82).

### Care of pediatric patients

DCs and NDs treat pediatric patients of all ages, newborn to adolescents. In both years, participating DC’s percentage of children and youth in their practice (11.5% in 2004, 12.5% in 2014) was half that of the responding NDs (22% in 2004, 23% in 2014) (*p* < 0.001). DCs estimated they see more pediatric patients per week (15.0 (SD 20.0), in 2004, 15.3 (SD 19.2) in 2014) compared to NDs (4.0 (SD 4.0) in 2004, 5.5 (SD 5.6) in 2014); DCs also see more patients overall: in 2014 they averaged 131 (SD 81) patients per week relative to the ND’s average 23 (SD 18) patients per week. In 2014, ND visits are significantly longer in duration (mean 46 min (SD 29, range 1 to 90) for first visit and 24 min follow up (SD 19, range 1 to 60)) relative to DC visits (29 min (SD 17, range 1 to 90) for first visit, 10 min for follow up (SD 8, range 1 to 60)) (*p* < .001).

**Fig. 1:**
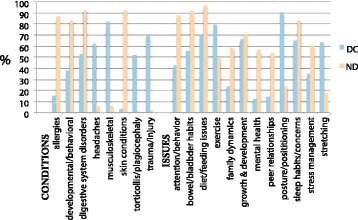
Top conditions and issues seen by DCs and NDs, 2014

DCs and NDs see a variety of common pediatric health conditions and issues. Figure 1 shows the conditions seen or issues addressed by at least 50% of either the DCs or NDs for 2014.

### Modification of treatment for pediatric patients

Almost all practitioners stated they modified their treatment approaches for children and youth: DCs: 95.0% in 2004, 75.3% in 2014; NDs: 95% in 2004, 84% in 2014. To better understand modification choices, in 2014, treatment modification was split into the five age categories, with a “do not treat” option for each category. The vast majority of practitioners modify treatments for newborns (DCs: 90.6%, NDs 88%), infants (DCs 91.5%, NDs 92%), and preschool-aged patients (DCs 89.7%, NDs 96%), the extent of the modification decreasing as the age of the patient approaches adulthood (adolescent: DCs 33.8%, NDs 55%). Common modifications in younger children were DCs using minimal force and light touch, NDs fewer herbs and lower doses; by adolescence, modifications were minimal relative to adult-style treatment. Similarly, the number of respondents choosing not to treat based on a child’s age dwindles as the child’s age increases: 7.1% of DCs and 8% NDs will not treat newborns, decreasing to 0% for preschool-aged and older children (NDs) and 0% for school-aged and older children (DCs).

### Other practice features

NDs commonly use NHPs, which include vitamins, minerals, probiotics, essential fatty acids, botanicals, etc. NDs both recommend NHPs (92% in 2004, 70% in 2014, *p* < .001) and sell NHPs (91% in 2004, 61% in 2014, p < .001). DCs also recommend NHPs to pediatric patients (32.1% in 2004, 34.1% in 2014, *p* = .65) and sell them (48.0% in 2004, 17.1% in 2014, p < .001).

In 2014, 67.9% of responding DCs indicated at least some use of x-rays with their pediatric patients. Of those, 47.6% indicated they regularly used x-rays on 5% or less of their pediatric patients; an additional 11.8% indicated that they would use x-rays only for specific clinical situations.

### Practitioner behaviour towards pediatric care

Case studies were used to assess DC and ND treatment and referral behaviours. Response details for all case studies are in Table 3.

**Table 3:** Case scenarios

			Chiropractic Doctors (DC)			Naturopathic Doctors (ND)			DC vs. ND
			2004	2014	2004 vs. 2014	2004	2014	2004 vs. 2014	
Case 1: Colitis	Their care will help the patient. n (%)	Not at all	7 (5)	19 (5.6)	*p* = .71^**a**^	0	0	*p* = .54^**a**^	**2004:** ***p*** **< .001** ^**a**^ **2014:** ***p*** **< .001** ^**a**^
		somewhat	64 (43)	132 (38.9)		25 (16)	13 (13)		
		very much	79 (53)	188 (55.5)		127 (84)	83 (87)		
	Average number of office visits needed for improvement. mean (SD) / range		4 (3)	4.3 (2.9)	*p* = .31^**b**^	2 (1)	2.0 (0.9)	*p* = .87^**b**^	**2004:** ***p*** **< .001** ^**b**^ **2014:** ***p*** **< .001** ^**b**^
			0–18	0–21		0–8	0–6		
	Patient will benefit from concurrent treatment. n (%)	Not at all	52 (34)	112 (34.0)	*p* = .082^**a**^	42 (30)	39 (42)	*p* = .11^**a**^	2004: *p* = .054^**a**^ 2014: *p* = .30^**a**^
		somewhat	85 (56)	162 (49.2)		73 (51)	44 (47)		
		very much	14 (9)	55 (16.7)		27 (19)	11 (12)		
	Would refer patient to another practitioner. n (%)	yes	76 (52)	215 (64.0)	***p*** **= .017** ^a^	95 (63)	57 (60)	*p* = .60^a^	2004: *p* = .058^a^ 2014: *p* = .48^a^
		no	69 (48)	121 (36.0)		55 (37)	38 (40)		
Case 2: Otitus media	Their care will help the patient. n (%)	Not at all	0^**c**^	53 (15.5)	–	0	0	*p* = .64^**a**^	2004: – **2014:** ***p*** **< .001** ^**a**^
		somewhat		131 (38.3)		6 (4)	5 (5)		
		very much		158 (46.2)		147 (96)	92 (95)		
	Average number of office visits needed for improvement. mean (SD) / range		0^**c**^	3.8 (2.9)	–	2 (1)	1.6 (0.7)	***p*** **= .002** ^**b**^	2004: – **2014:** ***p*** **< .001** ^**b**^
				0–21		0–5	0–5		
	Patient will benefit from concurrent treatment. n (%)	Not at all	0^**c**^	46 (13.6)	–	20 (15)	24 (25)	***p*** **= .008** ^**a**^	2004: – **2014:** ***p*** **< .001** ^**a**^
		somewhat		148 (43.9)		84 (61)	62 (65)		
		very much		143 (42.4)		34 (25)	10 (10)		
	Would refer patient to another practitioner. n (%)	yes	0^**c**^	273 (81.7)	–	77 (52)	56 (58)	*p* = .31^a^	2004: – **2014:** ***p*** **< .001** ^a^
		no		61 (18.3)		72 (48)	40 (42)		
Case 3: Febrile neonate	Their care will help the patient. n (%)	Not at all	42 (27)	135 (41.4)	***p*** **= .005** ^**a**^	26 (18)	31 (33)	***p*** **< .001** ^**a**^	2004: *p* = .095^**a**^ **2014:** ***p*** **< .001** ^**a**^
		somewhat	77 (49)	118 (36.2)		70 (49)	59 (62)		
		very much	38 (24)	72 (22.1)		48 (33)	5 (5)		
	Average number of office visits needed for improvement mean (SD) / range		0^d^	1.4 (2.1)	–	0^d^	0.4 (0.8)	–	2004: – 2014: ***p*** ** < .001** ^**b**^
				0–21			0–5		
	Patient will benefit from concurrent treatment. n (%)	Not at all	10 (6)	31 (9.5)	*p* = .49^**a**^	2 (1)	2 (2)	*p* = .15^**a**^	**2004:** ***p*** **= .043** ^**a**^ **2014:** ***p*** **< .001** ^**a**^
		somewhat	26 (17)	54 (16.6)		19 (13)	5 (5)		
		very much	122 (77)	241 (73.9)		128 (86)	88 (93)		
	Would refer patient to another practitioner. n (%)	yes	150 (95)	290 (91.5)	*p* = .17^a^	153 (98)	94 (99)	*p* = .59^a^	2004: *p* = .13^a^ **2014:** ***p*** **= .011** ^a^
		no	8 (5)	27 (8.5)		3 (2)	1 (1)		

a: chi-squared tests. b: t-tests. c: In 2014, the DC case study was changed to match the ND case study for comparison between the professions. d: In 2004, this question was not asked. Bold items are statistically significant

### Case study 1: colic

Most practitioners considered this to be colic. When asked if their professional care would help the patient, 2004 and 2014 within-profession responses were similar. DC practitioners’ primary treatment focus (314 respondents) would be to use spinal manipulation (78.3%) if physical assessment suggests utility, diet changes (14.6% for child, 6.1% for mom if breast feeding), and massage (16.9%). ND practitioners (95 respondents) would assess and treat primarily with diet changes (62% for child including prescribing probiotics; 48% for mom if breast feeding), homeopathy (46%), weak herbal or tea preparations (19%), and use topical castor oil (packs or massage) (18%). In 2014, 65.9% of DCs and 59% of NDs believe (somewhat or very much) that concurrent treatment by a medical practitioner would be of benefit; 64.0% of DCs and 60% of NDs would refer the patient to another health care practitioner (practitioner type not specified).

### Case study 2: acute otitis media

In 2014, almost all practitioners identified this as otitis media (in 2004, the DCs had a profession-specific question); DCs were more cautious about the value of their care for it relative to the NDs (DCs, 46.2% care will help patient very much, NDs, 95%). For treatment, DCs would primarily use spinal manipulation (98.5%) if indicated after assessment, massage (19.5%), dietary modifications (17.6%), and 3.8% would specifically refer to an MD for an antibiotic prescription. ND-preferred treatments were NHP products (79%), dietary modifications (66%), ear drops (60%), homeopathic remedies (18%), and 10% would prescribe antibiotics right away or after a few days. In 2014, 86.3% of DCs and 75% of NDs believe the patient would benefit (somewhat or very much) from concurrent treatment by a conventional medical practitioner; 81.7% of DCs and 58% of NDs would refer the patient to another health care provider.

### Case study 3: febrile neonate

When asked about a 3 week old febrile neonate, most practitioners would refer for emergency medical assessment: DCs: 94.9% in 2004 and 93.1% in 2014, and NDs: 98% in 2004 and 99% in 2014. Despite this, in 2014, only 73.9% DCs and 93% of NDs felt the patient would benefit (somewhat or very much) from concurrent treatment from a conventional medical practitioner. Additionally, in 2014, 58.3% of DCs and 67% of NDs felt their care, concurrent or follow-up, could help.

## Discussion

This study explored, over a decade, two popular complementary therapy professions in Canada regarding their pediatric training and care. Respondents reported a desire for greater pediatric education during their programs, with few considering their training adequate. For both professions, comfort in treating children increased with years of experience and with the age of the child.

Many DCs and NDs see infants, children and youth for a broad array of health concerns. Research evaluating the benefits and risks associated with DC and ND care of pediatric conditions, as well as the co-management of care with other health professions, is needed. The three case studies show that there is diverse practitioner opinion on treatment options, with non-standardized approaches to treatment of conditions (when to refer, when and how to treat, when to co-manage), likely reflecting variation in training, experience, and local treatment practices, an issue for many health care professions [11, 12].

Little work has been done evaluating pediatric care in complementary therapy practitioner offices. Work published by Lee and Kemper in 2000 had some similarities, such as roughly the same percentage of pediatric patients in their practice (DCs 11%, NDs 19%) [7, 8]. However, when asked a comparable question about a febrile neonate, our respondents were significantly more likely to refer to a physician. This is an important advance, and may reflect differences in training since Lee and Kemper’s work. Lee and Kemper reported that nearly all NDs reported treating children, but less than half had any formal pediatric training [7]. Our research suggests that little advance has been made in 15 years; most of our respondents sought additional pediatric training post-graduation. Many respondents noted that few undergraduate opportunities exist for clinical pediatric training experience, an issue likely underlying the very low training adequacy ratings.

A few participants did not refer the febrile neonate for emergency assessment. The first priority of education and care should be child safety and well-being, for any condition seen. Such essential knowledge and training should arise from core pediatric curriculum (lectures and hands-on experience) in DC and ND training programs, not principally from voluntary, non-standardized post-graduate courses, practice experience, or other local factors. We therefore suggest that DC and ND colleges review their hands-on clinical training, particularly for core essential pediatric conditions and “red flags” (i.e., potentially serious medical conditions with time sensitive need for recognition and management), in order to promote patient safety and increase comfort in care provision. Core concepts in pediatrics, as well as innovative approaches to teaching pediatrics and gaining clinical experience, are extensively developed and incorporated in the training programs for other health care professions. Outreach and collaboration between professions is recommended.

Like all research, our work has limitations. (1) We had a low rate of survey response, particularly in 2014, suggesting that our results may not be generalizable to other settings or populations. In 2014, organizational representatives from both professions mentioned survey fatigue, and some respondents questioned our motives for doing the survey. Respondents may have a particular interest in pediatric care, though there was no difference between the demographics of practitioners who did and did not see pediatric patients. Additionally, the 2014 DC demographics for age, years in practice, and size of practice community were similar between our survey respondents and published profession demographics, suggesting a high degree of similarity to the broader DC population; ND data were not available for comparison [13]. Regardless of the low response rate, most respondents in both surveys indicated their pediatric training was insufficient to meet their needs in clinical practice and wanted more training, strongly suggesting curricular enhancements need to be considered. (2) Respondents may have tried to portray their pediatric knowledge, skills, and attitudes in a favourable light. While this is possible, we noted very candid responses about their desire for additional pediatric training and how they would treat the children presented in the case scenarios.

## Conclusions

This study highlights important findings in Canadian DCs and NDs knowledge, attitudes, and behaviour towards children and youth in their care. The diversity of therapeutic approaches identified reflect the need to promote pediatric research on the benefits and risks of complementary therapies. There is a need to enhance pediatric training to address gaps identified by practitioners; emphasis should be given to conditions that would enhance patient safety. We call for greater collaboration between conventional and complementary therapy educational institutions to share core pediatric curriculum about conditions that could harm children if not recognized, to help future health care providers of all disciplines meet the needs of children in their care.

## Abbreviations

CI: Confidence interval
DC: Chiropractic doctor
ND: Naturopathic doctor
NHP: Natural health product
OR: Odds ratio
SD: Standard deviation
